# Somatostatin-based radiotherapy with [^90^Y-DOTA]-TOC in neuroendocrine tumors: long-term outcome of a phase I dose escalation study

**DOI:** 10.1186/1479-5876-11-17

**Published:** 2013-01-15

**Authors:** Nicolas Marincek, Ann-Catherine Jörg, Philippe Brunner, Christian Schindler, Michael T Koller, Christoph Rochlitz, Jan Müller-Brand, Helmut R Maecke, Matthias Briel, Martin A Walter

**Affiliations:** 1 Institute of Nuclear Medicine, University Hospital Basel, CH; 2 Swiss Tropical and Public Health Institute, University of Basel, CH; 3 Basel Institute for Clinical Epidemiology and Biostatistics, University Hospital Basel, CH; 4 Department of Oncology, University Hospital Basel, CH; 5 Division of Radiological Chemistry, University Hospital Basel, CH; 6 Department of Clinical Epidemiology and Biostatistics, McMaster University, Hamilton, CA; 7 Department of Molecular and Medical Pharmacology, David Geffen School of Medicine, UCLA, Los Angeles, USA

**Keywords:** Radiopeptide therapy, DOTATOC, DOTA-TOC, Survival, Neuroendocrine tumor

## Abstract

**Background:**

We describe the long-term outcome after clinical introduction and dose escalation of somatostatin receptor targeted therapy with [^90^Y-DOTA]-TOC in patients with metastasized neuroendocrine tumors.

**Methods:**

In a clinical phase I dose escalation study we treated patients with increasing [^90^Y-DOTA]-TOC activities. Multivariable Cox regression and competing risk regression were used to compare efficacy and toxicities of the different dosage protocols.

**Results:**

Overall, 359 patients were recruited; 60 patients were enrolled for low dose (median: 2.4 GBq/cycle, range 0.9-7.8 GBq/cycle), 77 patients were enrolled for intermediate dose (median: 3.3 GBq/cycle, range: 2.0-7.4 GBq/cycle) and 222 patients were enrolled for high dose (median: 6.7 GBq/cycle, range: 3.7-8.1 GBq/cycle) [^90^Y-DOTA]-TOC treatment. The incidences of hematotoxicities grade 1–4 were 65.0%, 64.9% and 74.8%; the incidences of grade 4/5 kidney toxicities were 8.4%, 6.5% and 14.0%, and the median survival was 39 (range: 1–158) months, 34 (range: 1–118) months and 29 (range: 1–113) months. The high dose protocol was associated with an increased risk of kidney toxicity (Hazard Ratio: 3.12 (1.13-8.59) vs. intermediate dose, p = 0.03) and a shorter overall survival (Hazard Ratio: 2.50 (1.08-5.79) vs. low dose, p = 0.03).

**Conclusions:**

Increasing [^90^Y-DOTA]-TOC activities may be associated with increasing hematological toxicities. The dose related hematotoxicity profile of [^90^Y-DOTA]-TOC could facilitate tailoring [^90^Y-DOTA]-TOC in patients with preexisting hematotoxicities. The results of the long-term outcome suggest that fractionated [^90^Y-DOTA]-TOC treatment might allow to reduce renal toxicity and to improve overall survival.

**Trial registration:**

ClinicalTrials.gov number:NCT00978211

## Background

**Figure 1:**
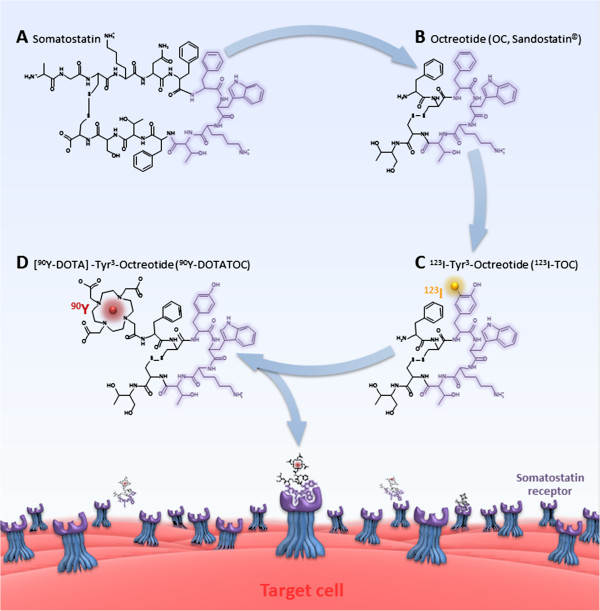
**The natural somatostatin receptor ligand, the 14 amino acid peptide somatostatin (A), was abridged to the biologically more stable 8 amino acid peptide Octreotide (OC, B), which is used for the treatment of symptomatic neuroendocrine tumors.** Introduction of a tyrosine into the 3rd position of the Octreotide sequence resulted in Tyr^3^-Octreotide (TOC, **C**), which allows for iodination of the tyrosine residue with the γ-emitter ^123^I and subsequent somatostatin receptor targeted imaging. For the use in somatostatin receptor targeted radiotherapy TOC was coupled with the chelator DOTA, which led to the study drug, the octapeptide DOTA-TOC (**D**).

Neuroendocrine tumors comprise a spectrum of different malignancies with a yearly increasing incidence and prevalence [1]. Most of these tumors express subtypes of the somatostatin receptor [2], which permits binding and internalization of the natural ligand, the 14 amino acid peptide somatostatin (Figure 1A). Stepwise modifications of the somatostatin sequence led to the development of the biologically more stable 8 amino acid somatostatin analogue Octreotide [3,4] (OC, Figure 1B), which nowadays has become a valuable tool for the treatment of symptomatic neuroendocrine tumors [5,6].

Introduction of a tyrosine into the third position of the Octreotide sequence resulted in Tyr^3^-Octreotide (TOC, Figure 1C), which allowed for iodination of the tyrosine residue with the γ-emitter ^123^I and subsequent somatostatin receptor targeted imaging [7]. The success of somatostatin receptor targeted imaging finally provided the rational for somatostatin receptor targeted radiotherapy.

For the use in somatostatin receptor targeted radiotherapy we coupled TOC with the chelator DOTA, which led to the octapeptide DOTA-TOC [8] (Figure 1D). DOTA-TOC can be labeled with various radioisotopes, including the γ-emitter ^111^In and the β-emitters ^177^Lu and ^90^Y. In an *in-vivo* treatment model ^90^Y-DOTA]-TOC was able to completely eliminate somatostatin receptor expressing xenografts [9]. Based on these promising *in-vivo* results we introduced ^90^Y-DOTA]-TOC into the clinic within a phase I dose escalation study.

The present work reports the long-term outcome of the dose escalation study with [^90^Y-DOTA]-TOC in patients with progressive neuroendocrine tumors.

## Subjects and methods

### Patients

We included patients (*i*) older than 18 years with (*ii*) histologically confirmed neuroendocrine tumors, (*iii*) metastasized disease, (*iv*) disease progression within 12 months before study entry and (*v*) visible tumor uptake in the pre-therapeutic somatostatin receptor scintigraphy. We excluded patients with (*i*) concurrent anti-tumor treatment, (*ii*) pregnancy, (*iii*) breast-feeding, (*iv*) urine incontinence, (*v*) preexisting hematological toxicities grade 3/4, and (*vi*) severe concomitant illness. The study was designed and carried out in accordance with good clinical practice guidelines, Swiss drug laws and the Declaration of Helsinki. The study was approved by the local ethics committee for human studies (EKBB-Reference-No.: M120/97; http://www.ekbb.ch) and registered (*ClinicalTrials.gov number NCT00978211*)*.* Written informed consent was obtained from all participants.

### Treatment

DOTA-TOC was synthesized in a five step synthetic procedure according to *Good Laboratory Practice*[8]. Radiolabeling was performed with the *β*-emitter ^90^Y for therapeutic purpose and 111 MBq of the *γ*-emitter ^111^In for intratherapeutic imaging [10,11]. Quality control was performed using solid phase extraction and high performance liquid chromatography, with a minimum required labeling yield >99.5%.

To the best of our knowledge, at the start of patient recruitment no published report was available on systemic applications of an ^90^Y labeled peptide into humans. Consequently, the activity for the initial application of ^90^Y-DOTA]-TOC was based on dosimetric assumptions as previously described [8]: (*i*) Based on the biodistribution of radiolabeled somatostatin analogues in imaging studies the kidneys were assumed to be the dose limiting organs for ^90^Y-DOTA]-TOC application. (*ii*) Based on dosimetric studies using a kidney phantom the kidney dose was assumed to be 1 Gy per 0.148 GBq of ^90^Y-DOTA]-TOC.

In the first arm (*Low Dose Group*), patients were treated with 4 initial cycles of escalating ^90^Y-DOTA]-TOC activities from 0.925 to 3.9 GBq, resulting in estimated kidney doses from 6.25 Gy to 25 Gy, which equals 25% to 100% of the maximum tolerated kidney dose in external beam irradiation [12]. As grade 3 hematotoxicities already occurred with this initial dose protocol and as severe hematotoxicities are potentially life-threatening, the further escalation steps were performed adapted to the body surface until the occurrence of grade 4 hematotoxicities. In the second arm (*Intermediate Dose Group*), patients were treated with 4 initial cycles of 1.85 GBq/m^2^ body surface ^90^Y-DOTA]-TOC, equating the maximum activities applied in the first arm. Finally, in the third arm (*High Dose Group*), the number of initial cycles was reduced from 4 to 2 using twice the ^90^Y-DOTA]-TOC activity used in the second arm, 3.7 GBq/m^2^ body surface.

After these initial cycles, further cycles were performed in case of (*i*) stabilization or decrease in the sum of the longest diameters of all pre-therapeutically detected tumor lesions, (*ii*) improvement in at least one of the five key symptoms: flush, diarrhea, pain, fatigue and involuntary weight-loss or (*iii*) post-therapeutic marker decrease after pre-therapeutic marker increase, as previously described [13]. For these further treatment cycles, patients crossed over to the actual dose escalation step at the time of re-treatment.

The use of Hartmann HEPA solution containing 10.7 g/L arginine and 6.9 g/L lysine and later NaCl containing 20.7 g/L arginine and 20.0 g/L lysine was introduced during the low dose protocol and was then continued during the intermediate and the high dose protocol. The solutions were applied before and after ^90^Y-DOTA]-TOC injection to inhibit tubular re-absorption of the radiopeptide [10,13,14]. The intratherapeutic biodistribution of the radiopeptide was imaged and tumor as well as kidney uptake were scored using a 4-point scale as previously described [13,15]: no uptake (*score 0*), uptake lower than liver uptake (*score 1*), uptake similar to liver uptake (*score 2*) and uptake higher than liver uptake (*score 3*). Patients were hospitalized 3 days for each cycle in accordance with the Swiss requirements for legal radiation protection.

### Follow-up

During hospitalization, clinical status and vital signs were monitored before and for 72 hours following each therapeutic application. All toxicities were continuously recorded. After discharge, blood chemistry and hematologic parameters were measured in biweekly intervals for 10 weeks after each cycle or until normalization of marker levels.

Further cycles were withheld because of progression, permanent toxicity, loss of the ability to travel to the treatment center or denial of further treatment. At this point, the follow-up was performed to obtain information on survival and long-term toxicities until the patient’s death. Follow-up data were obtained from the referring centers, with a minimum frequency of 2 follow-up visits per year, adapted to the patient’s individual requirements. All follow-up data were centrally collected and each case was reviewed and approved for completeness at the study center. Family physicians and the patients were directly contacted if additional follow-up results were needed.

Acute and long-term adverse events were graded according to the Common Terminology Criteria for Adverse Events version 3.0 (http://ctep.cancer.gov/protocolDevelopment/electronic_applications/docs/ctcaev3.pdf) of the National Cancer Institute. The kidney function was assessed using the *Modification of Diet in Renal Disease* (MDRD) formula [16]; renal toxicities were classified according to guidelines of the *National Kidney Foundation* (http://www.kidney.org). Severe renal toxicity was defined as toxicity grade 4 or 5 (glomerular filtration rate <30 or <15 mL/min/1.73 m^2^).

### Outcomes and Statistical Analyses

The main outcomes for this dose escalation study were hematological and renal toxicities. Predictors of hematological toxicities were investigated using logistic regression with the following pre-specified prognostic variables: gender, age, histology, duration of disease, previous surgery vs. no surgery, previous chemotherapy vs. no chemotherapy, previous radiation vs. no radiation, solitary metastases vs. multiple metastases, liver metastases vs. no liver metastases, bone metastases vs. no bone metastases, tumor uptake score and applied [^90^Y-DOTA]-TOC activity.

In order to accurately investigate predictors of renal toxicity the competing risk of death prior to renal toxicity was considered in all analyses. Cumulative incidence functions were used to display the proportion of patients with renal toxicity or the competing event of death as time progressed [17] and a *Cause-specific Cox regression model* for the subdistribution hazard [18] was employed. The following pre-specified variables were tested in the model: gender, age, baseline glomerular filtration rate, cumulative applied ^90^Y-DOTA]-TOC activity and treatment with low or high vs. intermediate doses of ^90^Y-DOTA]-TOC. Survival was not an *a priori* study endpoint; however, in a post hoc analysis we also analyzed the survival in the three groups from time of first ^90^Y-DOTA]-TOC treatment to death from any cause.

Predictors of survival were studied by multivariable Cox regression with the pre-specified candidate variables used for analyses of all hematotoxicities as described above, adjusted to the cumulative applied [^90^Y-DOTA]-TOC activity.

Sensitivity analyses were performed to assess the influence of the year of treatment and the influence of all pre-specified predictors on the 1-, 2- and 5-years survival, respectively. Two-sided *p* values of <0.05 were considered to indicate statistical significance.

## Results

### Patients

**Figure 2:**
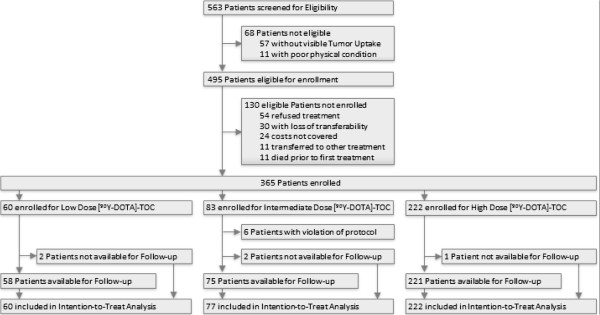
Patient flow.

A total of 563 patients were screened for eligibility, 68 patients (12.1%) were not eligible, 130 patients (23.1%) were eligible but not treated with [^90^Y-DOTA]-TOC. The last 6 patients (1.1%) of the intermediate dose protocol had received 2 cycles according to the intermediate dose protocol and then 2 cycles according to the high dose protocol; due to this violation of protocol they were excluded from all further analyses (Figure 2). The baseline characteristics of the remaining 359 patients are shown in Table 1. Generally, patients receiving low dose [^90^Y-DOTA]-TOC treatment in group 1 had the highest frequency of characteristics for advanced disease compared to patients recruited in succeeding groups, e.g. multiple metastases (95.0% vs. 85.7% and 87.4%) or bone metastases (26.7% vs. 22.1% and 21.2%, Table 1).

**Table 1:** **Baseline characteristics (*n = 359*)**

**Characteristic**		**Low dose (*n = 60*)**	**Intermediate dose (*n = 77*)**	**High dose (*n = 222*)**
Gender	Females	27 (45.0%)	30 (50.6%)	98 (44.1%)
	Males	33 (55.0%)	47 (49.4%)	124 (55.9%)
Age [y]	Median	51.1	55.4	59.0
	Range	18.1-77.0	21.9-80.5	20.5-81.1
Disease Duration [y]	Median	1.9	1.4	1.9
	Range	0.1-25.8	0.1-18.6	0.1-27.5
Pretreatment	Surgery	35 (58.3%)	36 (46.8%)	125 (56.3%)
	Chemotherapy	14 (23.3%)	16 (20.8%)	38 (17.1%)
	Radiation	21 (35.0%)	31 (40.3%)	72 (32.4%)
Extent	Single Metastasis	3 (5.0%)	11 (14.3%)	28 (12.6%)
	Liver Metastases	48 (80.0%)	62 (80.5%)	189 (85.1%)
	Bone Metastases	16 (26.7%)	17 (22.1%)	47 (21.2%)
Creatinine [μmol/L]	Median	63	71	69
	Range	22-258	26-138	33-369
Tumor Uptake	Score 1	5 (8.3%)	10 (13.0%)	18 (8.1%)
	Score 2	6 (10.0%)	8 (10.4%)	11 (5.0%)
	Score 3	49 (81.7%)	59 (76.6%)	193 (87.0%)
Kidney Uptake	Score 0	2 (3.3%)	3 (3.9%)	11 (5.0%)
	Score 1	4 (6.6%)	8 (10.4%)	20 (0.2%)
	Score 2	12 (20.0%)	10 (13.0%)	52 (23.4%)
	Score 3	42 (70.0%)	55 (71.4%)	137 (61.7%)
Histology	Carcinoid	19 (31.7%)	38 (49.4%)	106 (47.7%)
	PNET	15 (25.0%)	21 (17.3%)	64 (28.8%)
	Rare NET	17 (28.3%)	9 (11.7%)	26 (11.7%)
	Unknown Primary	9 (15.0%)	9 (11.7%)	26 (11.7%)

PNET: pancreatic neuroendocrine tumors, NET: neuroendocrine tumors, Rare NET include: medullary thyroid cancers, neuroblastomas, phaeochromocytomas, paragangliomas, small cell lung cancers and Merkel cell cancers.

### Group 1: Low Dose [^90^Y-DOTA]-TOC Treatment

**Figure 3:**
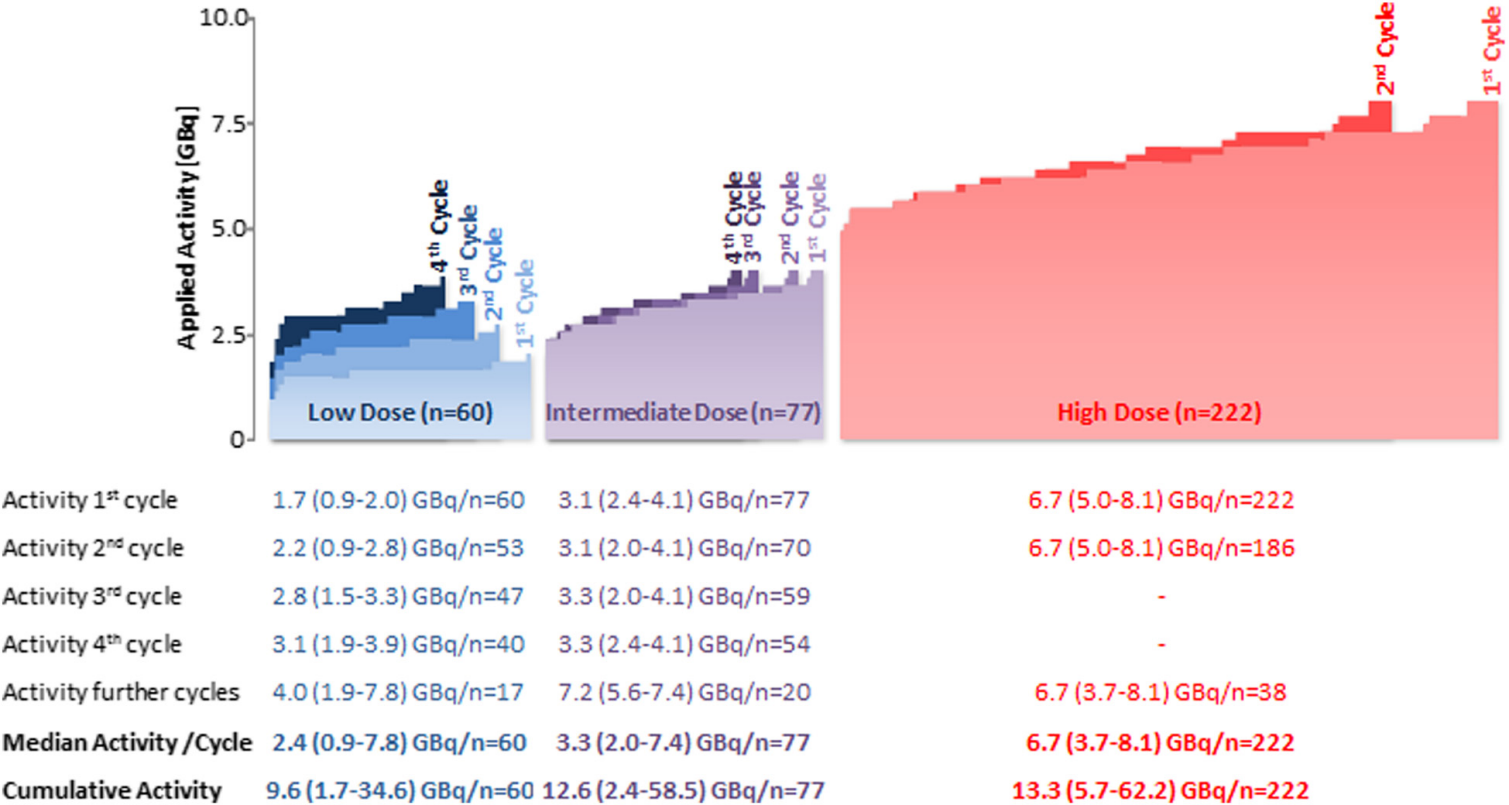
Dosage protocol.

A total of 60 patients were treated with 200 cycles according to the low dose protocol. The median activity per cycle was 2.4 GBq (range: 0.9-7.8 GBq), the median cumulative activity was 9.6 GBq (range: 1.7-34.6 GBq; Figure 3). Hematotoxicities developed in 39 patients (65.0%, Table 2): leukopenia grade 1–3 developed in 13 (21.7%), anemia grade 1–2 in 33 (55.0%) and thrombocytopenia grade 1–3 in 13 (21.7%) patients. Stabilization or decrease in tumor size was found in 11 (18.3%), clinical improvement in 7 (11.7%) and tumor marker decrease in 5 (8.3%) patients. A total of 17 patients (28.3%) received 28 further cycles according to the intermediate dose protocol (median cycle number: 4; range: 1–8). During a median follow-up of 15 (range: 1–158) months 1 patient (1.7%) developed grade 4 renal toxicity and 4 patients (6.7%) developed grade 5 renal toxicity. No grade 4 hematotoxicities were recorded and the subsequent patients were enrolled for the intermediate dose group.

**Table 2:** **Hematological toxicities (*n = 359*)**

**Low Dose (*n = 60*)**	***Total***	**Grade 1**	**Grade 2**	**Grade 3**	**Grade 4**
Leukopenia	*13 (21.7%)*	5 (8.3%)	6 (10%)	2 (3.3%)	0 (0%)
Anemia	*33 (55.0%)*	24 (40%)	9 (15%)	0 (0%)	0 (0%)
Thrombocytopenia	*13 (21.7%)*	8 (13.3%)	3 (5.0%)	2 (3.3%)	0 (0%)
**Intermediate Dose (*n = 77*)**					
Leukopenia	*18 (23.4%)*	9 (11.7%)	6 (7.8%)	3 (3.9%)	0 (0%)
Anemia	*43 (55.8%)*	35 (45.5%)	8 (10.4%)	0 (0%)	0 (0%)
Thrombocytopenia	*23 (29.9%)*	20 (26.0%)	2 (2.6%)	1 (1.3%)	0 (0%)
**High Dose (*n = 222*)**					
Leukopenia	*77 (34.7%)*	31 (14.0%)	31 (14.0%)	13 (5.9%)	2 (0.9%)
Anemia	*145 (65.3%)*	117 (52.7%)	22 (9.9%)	5 (2.3%)	0 (0%)
Thrombocytopenia	*92 (41.4%)*	68 (30.6%)	11 (5.0%)	6 (2.7%)	7 (3.2%)

All toxicities are scored according to the NCI criteria.

### Group 2: Intermediate Dose [^90^Y-DOTA]-TOC Treatment

A total of 77 patients were treated with 260 cycles according to the intermediate dose protocol. The median activity per cycle was 3.3 GBq (range: 2.0-7.4 GBq), the median cumulative activity was 12.6 GBq (range: 2.4-58.5 GBq; Figure 3). Hematotoxicities developed in 50 patients (64.9%, Table 2): leukopenia grade 1–3 developed in 18 (23.4%), anemia grade 1–2 in 43 (55.8%) and thrombocytopenia grade 1–3 in 23 (29.9%) patients. One patient developed acute myeloic leukemia after 4 cycles. Stabilization or decrease in tumor size was found in 21 (27.3%), clinical improvement in 13 (16.9%) and tumor marker decrease in 13 (16.9%) patients. A total of 20 patients (26.0%) received 31 further cycles according to the high dose protocol (median cycle number: 4; range: 1–10). During a median of 23 months (range: 1–118 months) of follow-up, 2 patients (2.6%) developed grade 4 renal toxicity and 3 patients (3.9%) developed grade 5 renal toxicity. No grade 4 hematotoxicities were recorded and the subsequent patients were enrolled for the high dose protocol.

### Group 3: High Dose [^90^Y-DOTA]-TOC Treatment

A total of 222 patients were treated with 408 cycles according to the high dose protocol. The median activity per cycle was 6.7 GBq (range: 3.7-8.1 GBq), the median cumulative activity was 13.3 GBq (range: 5.7-62.2 GBq; Figure 3). Hematotoxicities developed in 166 patients (74.8%, Table 2): leukopenia grade 1–4 developed in 77 (23.4%), anemia grade 1–3 in 145 (65.3%) and thrombocytopenia grade 1–4 in 92 (41.4%) patients. Stabilization or decrease in tumor size was found in 83 (37.4%), clinical improvement in 28 (12.6%) and tumor marker decrease in 29 (13.1%) patients. A total of 38 patients (17.1%) received 57 further cycles of [^90^Y-DOTA]-TOC, resulting in a median number of 2 (range: 1–8) cycles [^90^Y-DOTA]-TOC. During a median of 21 months (range: 1–113 months) of follow-up, 24 patients (10.8%) developed grade 4 renal toxicity and 7 patients (3.2%) developed grade 5 renal toxicity. Overall, grade 4 hematotoxicities were recorded in 6 patients (2.7%) and no further dose escalation was performed.

### Predictors of Hematotoxicity

**Figure 4:**
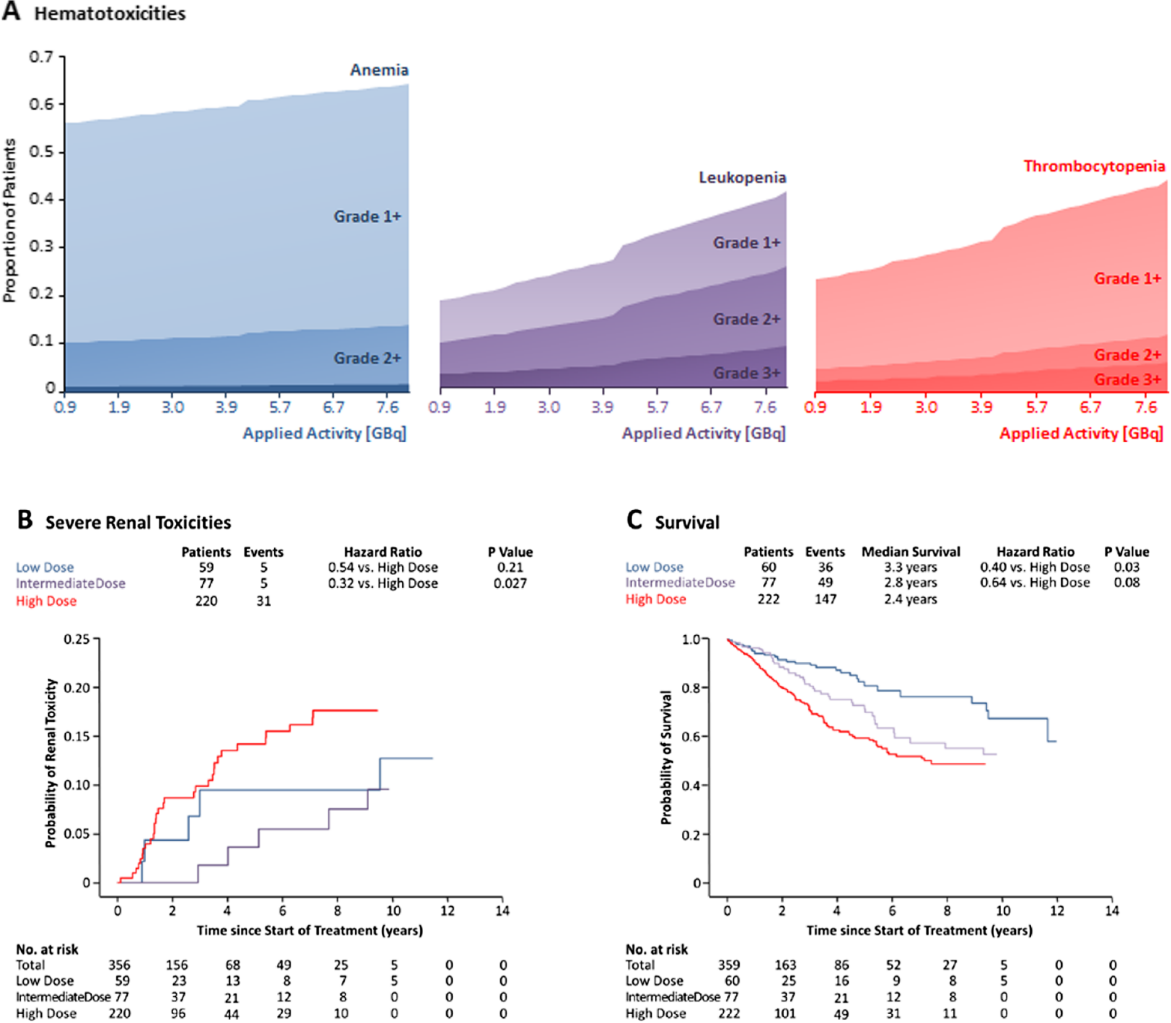
**Binary logistic regression plots displaying the frequency of anemia, leukopenia and thrombocytopenia at different therapeutic [**^**90**^**Y-DOTA]-TOC activities (A).** Cumulative incidence functions displaying the proportion of patients with renal toxicity are shown for low-dose, intermediate dose and high-dose [^90^Y-DOTA]-TOC treatment (**B**). Covariate-adjusted Kaplan-Meier estimates of overall survival are shown for low-dose, intermediate dose and high-dose [^90^Y-DOTA]-TOC treatment (**C**).

Higher frequencies of hematological toxicities were found at higher [^90^Y-DOTA]-TOC activities applied during a cycle (Figure 4A); however, these effects were not statistically significant for anemia (*p* = 0.58), leukopenia (*p* = 0.12) or thrombocytopenia (*p* = 0.17). One patient in the intermediate dose group developed acute myeloid leukemia.

### Predictors of Renal Toxicity

Three patients had a baseline glomerular filtration rate below 30 mL/min/1.73 m^2^; the remaining 356 patients were included in the competing risk analyses to identify predictors of kidney toxicity. These analyses suggested the highest risk of kidney toxicity at high dose [^90^Y-DOTA]-TOC treatment (Hazard Ratio: 3.12 (1.13-8.59) vs. intermediate dose, p = 0.027, Figure 4B). A further predictor for kidney toxicity was a low baseline glomerular filtration rate (Table 3).

**Table 3:** **Hazard ratios for overall survival and severe kidney toxicities (*n = 359*)**

**Covariate**		Hazard ratio (95% CI)*	**P Value**
**Overall survival**			
Gender	*(male vs. females)*	0.97 (0.74-1.28)	0.84
Age	*(per year)*	1.004 (0.99-1.02)	0.49
Duration of Disease	*(per year)*	0.98 (0.94-1.02)	0.30
Previous Surgery	*(vs. no surgery)*	0.62 (0.45-0.84)	0.002
Previous Systemic Therapies	*(vs. no systemic therapies)*	1.48 (1.08-2.01)	0.014
Previous Radiation	*(vs. no radiation)*	0.93 (0.64-1.36)	0.71
Single Metastasis	*(vs. multiple metastases)*	0.66 (0.26-1.35)	0.26
Liver Metastases	*(vs. no liver metastases)*	1.45 (0.86-2.44)	0.17
Bone Metastases	*(vs. no bone metastases)*	1.57 (1.10-2.23)	0.013
Tumor Uptake Score 2	*(vs. Uptake Score 1)*	0.70 (0.31-1.61)	0.40
Tumor Uptake Score 3	*(vs. Uptake Score 1)*	0.40 (0.18-0.91)	0.028
Low Dose [^90^Y-DOTA]-TOC	*(vs. High Dose)*	0.40 (0.17-0.93)	0.03
Intermediate Dose [^90^Y-DOTA]-TOC	*(vs. High Dose)*	0.64 (0.38-1.06)	0.08
**Severe Kidney Toxicity**			
Gender	*(male vs. females)*	0.62 (0.33-1.18)	0.14
Age	*(per 10 years)*	0.99 (0.96-1.02)	0.64
Baseline Glomerular Filtration Rate	*(per 10 mL/min/1.73 m*^*2*^*)*	1.23 (1.09-1.39)	0.001
Low Dose [^90^Y-DOTA]-TOC	*(vs. High Dose)*	0.54 (0.20-1.43)	0.21
Intermediate Dose [^90^Y-DOTA]-TOC	*(vs. High Dose)*	0.32 (0.11-0.88)	0.027

*Estimates for each co-variable have been adjusted for histology as a categorical co-variable, for the cumulative [^90^Y-DOTA]-TOC activity as well as for all other co-variables listed.

### Predictors of Survival

In the low dose group (median follow-up: 15 months, range: 1–158 months), 36 patients (60.0%) died, 22 patients (36.7%) survived and 2 patients (3.3%) were not available for follow-up. In the intermediate dose group (median follow-up: 15 months, range: 1–118 months), 49 patients (63.6%) died, 26 patients (33.8%) survived and 2 patients (2.6%) were not available for follow-up. In the high dose group (median follow-up: 15 months, range: 1–158 months), 147 patients (66.2%) died, 74 patients (33.3%) survived and 1 patient (0.5%) was not available for follow-up. The overall median survival was 39 (range: 1–158) months in the low dose group, 34 (range: 1–118) months in the intermediate group (HR: 1.60 (0.89-2.85) vs. low dose, p = 0.11) and 29 (range: 1–113) months in the high dose group (HR: 2.50 (1.08-5.79) vs. low dose, p = 0.03, Figure 4C, Table 3). The hazard ratios for the main predictors did not change significantly over the enrollment period. The predictors for the 1-, 2- and 5-years survival are listed in Table 4.

**Table 4:** **Hazard ratios for overall survival 1, 2 and 5 years after start of [**^**90**^**Y-DOTA]-TOC Therapy (*n = 359*)**

		**1-YEAR SURVIVAL**		**2-YEARS SURVIVAL**		**5-YEARS SURVIVAL**	
**Covariate**		Hazard ratio (95% CI)*	**P Value**	Hazard ratio (95% CI)*	**P Value**	Hazard ratio (95% CI)*	**P Value**
Gender	*(male vs. females)*	1.00 (0.61-1.63)	0.99	1.08 (0.75-1.57)	0.66	1.00 (0.75-1.35)	0.98
Age	*(per 10 years)*	1.00 (0.98-1.02)	0.69	0.99 (0.98-1.00)	0.19	1.00 (0.99-1.01)	0.83
Duration of Disease	*(per year)*	0.94 (0.86-1.04)	0.22	0.93 (0.86-0.99)	0.03	0.97 (0.93-1.01)	0.16
Previous Surgery	*(vs. no surgery)*	0.59 (0.33-1.04)	0.07	0.57 (0.38-0.87)	0.009	0.63 (0.45-0.87)	0.006
Previous Chemotherapy	*(vs. no chemotherapy)*	1.28 (0.75-2.19)	0.37	1.28 (0.86-1.93)	0.23	1.68 (1.21-2.32)	0.002
Previous Radiation	*(vs. no radiation)*	0.63 (0.31-1.31)	0.22	0.75 (0.45-1.25)	0.27	0.93 (0.62-1.39)	0.72
Solitary Metastases	*(vs. multiple metastases)*	0.38 (0.11-1.37)	0.14	0.60 (0.22-1.60)	0.31	0.63 (0.29-1.38)	0.25
Liver Metastases	*(vs. no liver metastases)*	1.83 (0.68-4.95)	0.24	1.40 (0.68-2.92)	0.36	1.57 (0.88-2.80)	0.13
Bone Metastases	*(v. no bone metastases)*	1.59 (0.90-2.81)	0.11	1.18 (0.74-1.87)	0.49	1.38 (0.94-2.01)	0.10
Tumor Uptake Score 2	*(vs. uptake score 1)*	1.47 (0.35-6.14)	0.60	2.10 (0.69-6.42)	0.19	0.77 (0.31-1.92)	0.58
Tumor Uptake Score 3	*(vs. uptake score 1)*	0.51 (0.13-2.09)	0.35	0.85 (0.28-2.61)	0.78	0.40 (0.16-0.98)	0.05
Low Dose [^90^Y-DOTA]-TOC	*(vs. High Dose)*	0.93 (0.19-4.58)	0.93	0.39 (0.13-1.19)	0.10	0.39 (0.16-0.96)	0.04
Intermediate Dose [^90^Y-DOTA]-TOC	*(vs. High Dose)*	0.74 (0.24-2.31)	0.61	0.52 (0.25-1.11)	0.09	0.57 (0.33-0.99)	0.05

*Estimates for each co-variable have been adjusted for histology as a categorical co-variable, for the cumulative [^90^Y-DOTA]-TOC activity as well as for all other co-variables listed.

## Discussion

The results of this dose escalation study suggest increasing hematological toxicities with increasing [^90^Y-DOTA]-TOC activities. Especially the high dose protocol was associated with a low but present risk of grade 4 hematotoxicities and a higher risk of kidney toxicities. Remarkably, the post hoc analyses suggested a potentially longer survival under the low dose protocol.

Hematotoxicity is an acute toxicity that can be due to irradiation from ^90^Y-DOTA]-TOC circulating through the body or binding to somatostatin receptors on bone marrow cells [19] or due to the small fraction of free ^90^Y that is administered during treatment cycles and that integrates into the bone matrix [20]. The present study suggests higher risk of anemia, leukopenia and thrombocytopenia at higher administered activities of ^90^Y-DOTA]-TOC. These results might represent a step towards preventing severe hematotoxicity by tailoring the applied ^90^Y-DOTA]-TOC activities, e.g. in patients with preexisting hematological toxicities.

Renal toxicity, on the other hand, is a late toxicity of ^90^Y-DOTA]-TOC. It is mainly due to the glomerular filtration and the tubular retention of the radiopeptide and its fragments and usually develops several months or years after treatment. Renal toxicity still represents the dose limiting toxicity in somatostatin based radiopeptide therapy. Renal toxicity rates between 0% and 24% have been reported for treatment with the radiopeptides ^90^Y-DOTA]-TOC and ^177^Lu-DOTA]-TOC [21-26]. These studies have used different inclusion criteria, especially regarding the baseline kidney function; they have used different treatment protocols, different follow-up schemes and different definitions of renal toxicity. Intra-study comparisons are highly warranted to allow for a comparison of the benefits and harms of both radiopeptides. The present results, however, suggest that fractionated low dose protocols might help to reduce this main toxicity of ^90^Y-DOTA]-TOC.

High dose ^90^Y-DOTA]-TOC therapy was introduced in our department in 1999 and had become the standard treatment regime, as the extent of short-term hematological and renal toxicities had shown to be acceptable [14]. The high dose protocol, in comparison to more fractionated dosage protocols, specifically allowed for a significant reduction of travelling efforts for patients recruited from Europe, North America, South America, Africa and Asia. Due to the systematic evaluation of the long-term outcome of all patients treated with ^90^Y-DOTA]-TOC [13] we were now able to also analyze the long-term outcome after the different dosage protocols. These present results reveal that fractionated low dose protocols might help to reduce the toxicity and improve the survival after ^90^Y-DOTA]-TOC.

Strengths of the present study include the comprehensive recruitment of 365 patients, the sufficiently long follow-up to detect renal toxicities months or years after [^90^Y-DOTA]-TOC treatment and the use of competing risk models to assess the long-term toxicities of [^90^Y-DOTA]-TOC.

The longer survival under the low dose protocol, nevertheless, represents an unexpected finding. However, in the present study survival was not a primary outcome. Furthermore, treatment allocation occurred in a non-randomized fashion, as naturally, the clinical introduction of a novel anti-cancer drug is done in a dose escalation study. Dose escalation studies may principally underestimate the outcome of patients receiving the initial dosage regime due to recruitment of very advanced cases to initially receive a new drug or due to advances in supportive care during the enrollment period. Still, in the present analysis, which was adjusted for all baseline characteristics and the cumulative administered activity, the longest survival was found for the initial dosage step. A randomized trial is warranted in order to confirm the results on survival and long-term toxicity of fractionated versus high dose ^90^Y-DOTA]-TOC therapy. Such a prospective trial should use the ECOG status [27], the ENETS grading system [28] and RECIST criteria [29] to investigate potential differences in response as well as differences in the time-to-progression at different dosage schemes. Furthermore, such a trial should examine a possible correlation of pre-existing diabetes, hypertension and proteinuria on kidney toxicity after ^90^Y-DOTA]-TOC.

In conclusion, herein we describe the outcome after clinical introduction and dose escalation of [^90^Y-DOTA]-TOC for somatostatin receptor targeted therapy in patients with metastasized neuroendocrine tumors. This study compares survival, acute toxicities and long-term toxicities of the different [^90^Y-DOTA]-TOC dosage protocols applied. Increasing [^90^Y-DOTA]-TOC activities may be associated with increasing hematological toxicity. The dose related hematotoxicity profile of [^90^Y-DOTA]-TOC could facilitate tailoring [^90^Y-DOTA]-TOC in patients with preexisting hematotoxicities. The results on the long-term outcome suggest that fractionated [^90^Y-DOTA]-TOC treatment might allow to reduce renal toxicity and to improve the overall survival.

## Competing interests

The authors declare that they have no competing interests.

## Authors’ contributions

NM, ACJ and PB were involved in designing the study; they collected all data and participated in drafting the manuscript. CS, MTK, JMB, HRM and MB designed the study, analyzed all data and critically revised the manuscript. MAW supervised study design, collection and analysis of all data and drafting of the manuscript. All authors read and approved the final manuscript.
